# Feel Your Reach: An EEG-Based Framework to Continuously Detect Goal-Directed Movements and Error Processing to Gate Kinesthetic Feedback Informed Artificial Arm Control

**DOI:** 10.3389/fnhum.2022.841312

**Published:** 2022-03-11

**Authors:** Gernot R. Müller-Putz, Reinmar J. Kobler, Joana Pereira, Catarina Lopes-Dias, Lea Hehenberger, Valeria Mondini, Víctor Martínez-Cagigal, Nitikorn Srisrisawang, Hannah Pulferer, Luka Batistić, Andreea I. Sburlea

**Affiliations:** ^1^Institute of Neural Engineering, Graz University of Technology, Graz, Austria; ^2^BioTechMed, Graz, Austria; ^3^RIKEN Center for Advanced Intelligence Project, Kyoto, Japan; ^4^Brain-State Decoding Lab, Albert-Ludwigs-Universität Freiburg, Freiburg, Germany; ^5^Stereotaxy and Functional Neurosurgery Department, Uniklinik Freiburg, Freiburg, Germany; ^6^Biomedical Engineering Group, E.T.S. Ingenieros de Telecomunicación, University of Valladolid, Valladolid, Spain; ^7^Biomedical Research Networking Center in Bioengineering, Biomaterials and Nanomedicine (CIBER-BBN), Valladolid, Spain; ^8^Faculty of Engineering, Department of Computer Engineering, University of Rijeka, Rijeka, Croatia

**Keywords:** electroencephalogram (EEG), brain-computer interface (BCI), goal-directed movement, movement detection, trajectory decoding, error-related potential, kinesthetic feedback, spinal cord injury (SCI)

## Abstract

Establishing the basic knowledge, methodology, and technology for a framework for the continuous decoding of hand/arm movement intention was the aim of the ERC-funded project “Feel Your Reach”. In this work, we review the studies and methods we performed and implemented in the last 6 years, which build the basis for enabling severely paralyzed people to non-invasively control a robotic arm in real-time from electroencephalogram (EEG). In detail, we investigated goal-directed movement detection, decoding of executed and attempted movement trajectories, grasping correlates, error processing, and kinesthetic feedback. Although we have tested some of our approaches already with the target populations, we still need to transfer the “Feel Your Reach” framework to people with cervical spinal cord injury and evaluate the decoders’ performance while participants *attempt* to perform upper-limb movements. While on the one hand, we made major progress towards this ambitious goal, we also critically discuss current limitations.

## Introduction

“Making the paralyzed move” is a dream for many researchers but even more for people suffering from a spinal cord injury (SCI) or other diseases leading to non-functional limbs and therefore a dramatic decrease in quality of life. While walking is always the first function an independent observer thinks is most critical, affected people usually have other wishes (Anderson, 2004). The higher the lesion in the spinal cord, the less important the walking. While very high lesions in the cervical spine lead to dysfunction of breathing and all motoric and sensory functions, a lesion in the lower cervical levels leads to restricted hand and arm movements, whereas breathing, speaking and head movements are usually not affected (Rupp, 2020). Besides, vegetative functions can also be reduced or affected.

Currently, there are not many options for the restoration of hand and arm function. Although there have been attempts by using functional electrical stimulation (FES) systems, controlled *via* movements of the contralateral shoulder (e.g., the implanted Freehand system, or research systems based on transcutaneous electrodes), there is no real system available for full arm function restoration (Eck and Rupp, 2021). Sometimes, surgical interventions like tendon or muscle transfers help to stabilize, e.g., hand rotation or improve biceps function (Dengler et al., 2021). On the other hand, there exist robotic arm systems, mounted on the wheelchair, which can be, although very cumbersome, controlled with, e.g., a chin joystick.

In neuroscience, the restoration of hand and arm function has been a research topic since the late 90s. Relatively soon, the ambition of “reading” the intention of movement from brain activity and transferring it into real movement with the help of a brain-computer interface (BCI) has emerged (Wolpaw et al., 2002; Millán et al., 2010; Wolpaw and Wolpaw, 2012; Brunner et al., 2015).

Recordings and real-time interpretation of neural activity originating within the motor cortex and other motor-related areas, first done in non-human primates (Georgopoulos et al., 1982; Kalaska et al., 1989) and later in humans, led to the first BCIs for the control of robotic arms. Up to 10 degrees of freedom could be successfully controlled by end-users with implanted multielectrode arrays (MEAs; Hochberg et al., 2006; Collinger et al., 2013; Wodlinger et al., 2015). Neuroprosthetic devices, i.e., systems based on functional electrical stimulation (FES; Rupp et al., 2015), applied to the upper limb of tetraplegic participants, can also be successfully controlled by invasive BCIs (Bouton et al., 2016; Ajiboye et al., 2017). While electrocorticogram (ECoG) is less invasive than MEAs, the first results for motor control applied in tetraplegics were recently reported (Silversmith et al., 2020).

The first application of a non-invasive BCI based on the electroencephalogram (EEG) to control the lost hand function of a high spinal cord injured male was presented in 2000 (Pfurtscheller et al., 2000). This and subsequent works relied on power modulations of sensorimotor rhythms associated with the imaginations of different limbs (Pfurtscheller and da Silva, 1999). Introducing functional electrical stimulation and neuroprosthetics led to more meaningful control (Pfurtscheller et al., 2003; Müller-Putz et al., 2005). In parallel, Scherer et al. (2004, 2008), Wolpaw and McFarland (2004), and McFarland et al. (2010) demonstrated 2D and 3D cursor control. Further developments such as hybrid BCIs (Pfurtscheller et al., 2010; Müller-Putz et al., 2011) and coding of brain patterns (Müller-Putz et al., 2010) showed small successes (Rohm et al., 2013). However, a non-invasive natural control of a full arm movement was at this point out of reach.

Back in 2010, Bradberry et al. (2010) demonstrated three-dimensional movement decoding in the center out tasks. Subsequently, several investigations were performed to explore the possibility to also decode movement trajectories from EEG (Lv et al., 2010; Ofner and Müller-Putz, 2012; Kim et al., 2015).

The long-term vision of our research is to realize a non-invasive EEG-based, intuitive controller for an upper extremity neuroprosthesis or robotic arm in people with high SCI. There are some works done in this direction, for example by Meng et al. (2016), who demonstrated that non-disabled people can perform tasks which require multiple degrees of freedom by a combination of two sequential low dimensional controls. They were using combinations of left and right-hand movement imaginations to achieve 2D control. Sequentially, they were then able to move a robotic arm up and down and grasp an object.

Back in 2015 when our project, “Feel Your Reach”, granted from the European Research Council (ERC) started, we could see that either trajectory decoding was possible to some extent, but only in offline scenarios, or unnatural control sequences were used to control a robotic arm, as described above.

The main idea of the project was twofold. First, we aimed to characterize several brain patterns and mechanisms that encode information about goal-directed movement intention, movement kinematics, error processing, and processing of kinesthetic sensory feedback in the EEG. Second, the mechanisms should be combined in a hybrid framework to derive a control signal that would allow an end-user to steer an artificial arm, a robotic limb, which can be mounted to the wheelchair, to a selected goal. Supported by the kinesthetic sensory feedback and continuous error detection, the user should be enabled to control the artificial arm efficiently in a natural way. The final aim of this project is to apply these methods to individuals with spinal cord injury.

This current article describes our findings and studies so far on all the aforementioned topics.

## Methodology

**Figure 1:**
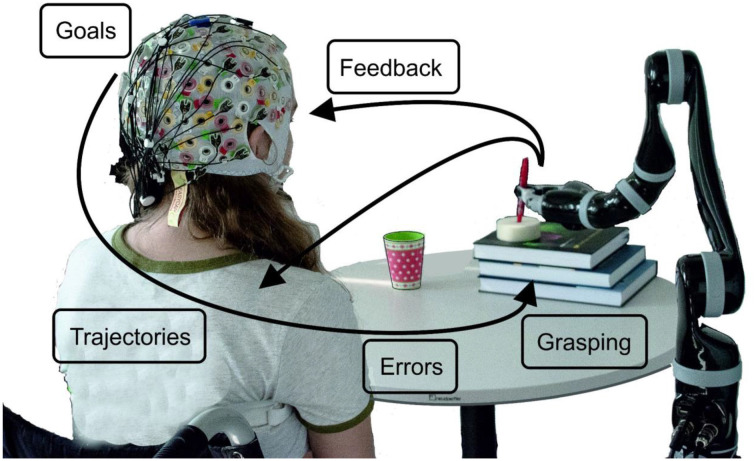
Schematic overview of the project ideas. The robot’s movement can start when a goal-directed movement intention is detected. By decoding trajectories the movement can be performed, always taking care, whether the detection of errors occurs. How grasping is reflected in neural patterns has been investigated as well as how visual and kinesthetic feedback influences the decoding accuracy.

In Figure 1 we illustrate the project idea and subsequently, we review a series of studies, which we performed and published over the last 6 years. Thus, we start with goal-directed movement planning and movement detection in an asynchronous classification scenario: (1) this is followed by our approach to trajectory decoding; (2) from offline studies to several online feedback experiments. While grasping is one logical end of a goal-directed arm movement, we investigated how a large variety of grasps are represented in neural patterns; (3) it is well known that BCIs always come along with wrong detections and misclassifications. Thus, we investigated neural correlates of errors during, e.g., robotic arm control, and used the detections to correct wrong movements; (4) one major goal of the project is to also include kinesthetic feedback additionally to visual feedback with the goal to advance trajectory decoding; and (5) furthermore, several methods to remove eye-dipole-induced artifacts from brain signals have been developed and evaluated. This was a crucial step, as the typical restrictions on eye movements imposed by current BCI protocols are also one reason why the control feels unnatural to the user. Robust eye-artifact correction models allow for the incorporation of visual guidance, an aspect which is critical in daily-life upper-limb movements.

### Goal-Directed Movement Planning and Detection

Studying the early stages of goal-directed movement planning can be of interest for BCIs which aim to restore or replace motor functions because the majority of such BCI applications involve interactions with targets. An important example of such an interaction would be the activation of a robotic arm to grasp a glass located in the vicinity of the user. The neural correlates of goal-directed movements and their differences from movements which do not result in an interaction with a particular goal have, over the past years, mainly been studied in movement observation tasks (Rizzolatti et al., 2014) and often using other neuroimaging techniques, like functional magnetic resonance imaging (fMRI). Studies show, for instance, a greater activation of the posterior parietal cortex (PPC) during the observation of goal-directed actions, when compared to the observation of the same actions but without a goal (Buccino et al., 2001). So, one of our primary objectives is to understand how goal-directed movements are represented in the EEG and how they differ from movements which do not have specific targets.

While the execution or imagination of motor tasks can be represented in power modulations in different frequency bands (mainly of sensorimotor rhythms, SMR) commonly exploited in SMR-based BCIs, low-frequency time-domain (LFTD) signals within the delta frequency range of the EEG can provide rich information about the users’ movement intentions and the characteristics of the upcoming movement. Concretely, movement-related cortical potentials (MRCPs) are LFTD potentials that are neural correlates of movement planning and execution and are time- and phase- locked to the movement onset (Kornhuber and Deecke, 1964; Shibasaki and Hallett, 2006). MRCPs features have been exploited for movement detection (i.e., asynchronous classification of movement vs. rest) and are characterized by a slow negative deflection before movement execution (ME), imagination (MI), or attempted ME, reaching the maximum negativity near the movement onset, followed by a positive rebound before returning to the baseline level. Over the last decade, MRCP features have been shown to be a rich alternative to power modulations since not only they can be exploited for movement detection (Niazi et al., 2011; Jochumsen et al., 2013, 2015; López-Larraz et al., 2014; Sburlea et al., 2015; Jiang et al., 2015; Liu et al., 2018), but also for classification of movement-related parameters like speed (Gu et al., 2009), force (Jochumsen et al., 2013), or even different types of grasps (Schwarz et al., 2018) and other upper-limb movements (Ofner et al., 2017). Importantly, our group has shown that different upper-limb movements can be decoded in individuals with complete SCI, and additionally that such decoding is possible online (Ofner et al., 2019). In a single-case online proof-of-concept, movement *detection* was also evaluated. A true positive rate of 30% was obtained for the detector, with more than three false positives per min (FP/min).

**Figure 2:**
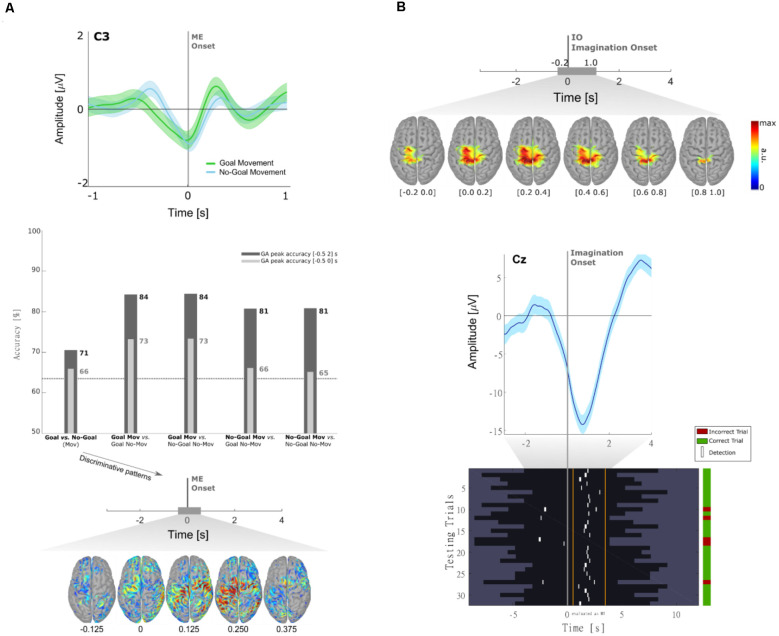
**(A)** Neural correlates of goal-directed movements [adapted from Pereira et al. (2017)]. Grand-average MRCPs (*n* = 10) over electrode C3 with respect to the ME onset and respective confidence interval, for both Goal and No-Goal conditions (*top panel*). Time-locked classification results: peak accuracies relative to the task pair comparisons for the time-windows before and after movement onset (*middle panel*). Discriminative spatial patterns in the source space for the Goal Movement vs. No-Goal Movement condition (*bottom panel*). **(B)** Detection of self-initiated goal-directed MIs of reach-and-grasp movements [adapted from Pereira et al. (2018)]. Grand-average MRCPs (*n* = 15) on the source space with respect to the imagination onset (top panel). MRCP on channel Cz of participant s01 time-locked to the imagination onset (*middle panel*). For that same participant, we show the single-trial image showing the imagination detections (marked in white) over each testing trial (*bottom panel*). A trial was considered when there was at least one true positive, and no false positives. On the group level, 53 ± 17% of trials were correctly classified (chance level of 20%).

In Pereira et al. (2017) we have shown that MRCPs differ between goal and non-goal-directed movements. In this study, a population of 10 non-disabled participants performed reach-and-touch movements with the same kinematics. Differences between the goal- and non-goal-directed conditions were found not only in the negative slope before movement onset on the central electrodes (Figure 2A, top panel) but also on the reafferent potential after movement onset. The results of an offline single-trial classification procedure showed that the performance of a movement detector, which exploited such MRCP features, was significantly higher when the movement was directed towards a goal (Figure 2A, middle panel). Notably, movement detection was possible before movement onset in all participants for the goal-directed movement (average accuracy of 73%), but in only 6 out of 10 participants for the non-goal-directed movement (average accuracy of 66%). These results suggest goal-directedness as a factor that can improve movement detection performance. Moreover, discrimination of goal movement vs. non-goal movement was possible with an average accuracy of 71%. When analyzing the correspondent classifier patterns (Figure 2A, bottom panel), we could determine that the brain areas relevant for goal vs. non-goal discrimination were the supplementary motor area, premotor cortex (PM), and superior parietal lobule, which constitute a fronto-parietal network and have been previously associated with movement goals (Saxe et al., 2004; Rizzolatti et al., 2014). It is fair to assume that for the goal-directed movement condition-specific information about the spatial location of the target had to be integrated for motor planning, which additionally explains the involvement of the parietal regions known for their role in visuomotor transformations (Andersen et al., 1997; Rizzolatti et al., 2014; Vingerhoets, 2014). After the movement onset, the discriminant patterns show activations not only in the primary motor (M1) but also in the left posterior parietal cortex (PPC), which is in consistency with the findings in Buccino et al. (2001) using fMRI.

A limitation of Pereira et al. (2017) and most of the studies on MRCPs for movement decoding and detection, is that the potentials are time-locked to the movement onset or to discrete cues. If one wants to exploit MRCPs in a population with little or no residual movement on their upper-limbs, as in complete cervical SCI, it is often not possible to time-lock to a movement onset. With the impossibility to measure a movement onset, which would be used to define a feature extraction window for the movement class, discrete “go-”cues are used as an alternative time-locking point. However, this strategy comes not only at the cost of the influence of external cues on the EEG signals but also with the fact that the movements are not self-initiated (or self-paced). As a consequence, it is unlikely that a movement detector trained on cue-based data would generalize to the envisioned scenario with self-initiated movements (Figure 1). In Pereira et al. (2018), we attempted to circumvent this challenge and allowed non-disabled participants to perform self-initiated goal-directed reach-and-grasp MI. The estimation of the MI onset was accomplished by introducing a scroller with numbers on a computer monitor, and instructing the participants to memorize the number that was on the scroller when they perceived the urge to perform the MI. We show for the first time that it is possible to extract the MRCPs features around a self-initiated MI onset to train a movement detector (Figure 2B). This detector was evaluated offline in an asynchronous manner, reaching an average percentage of correctly classified trials of 53%. Performance was above chance-level for all participants (around 20%). Note that this measure of performance does not allow for false positives within the trial period [more details in Pereira et al. (2018)]. This approach is theoretically transferable to movement attempts, which have been proven to be a good control strategy for SCI participants (Blokland et al., 2012). However, it is suboptimal to introduce an additional (memorization) task parallel to the imagination task. Not only is the overall task more complex, but it can also distract the participants from the main focus of the experiment which is the motor task itself. In fact, parallel to this work, Aliakbaryhosseinabadi et al. have shown that the higher attention diversion imposed by a dual-task (counting and moving) led to a significant reduction in specific MRCPs features, further affecting the movement detection performance when compared with the single movement task (Aliakbaryhosseinabadi et al., 2017).

**Figure 3:**
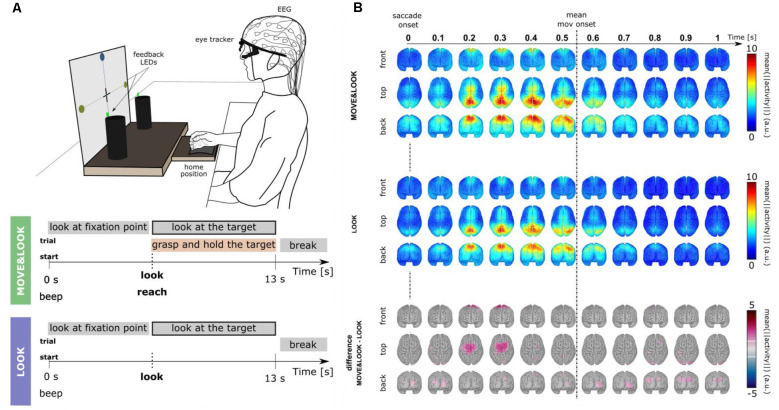
**(A)** Experimental paradigm [adapted Pereira et al. (2021)]. Participants performed two main conditions. In the *MOVE&LOOK* condition participants initially fixated their gaze on one of the points on the vertical board, and then freely decided when (within the 13 s) to perform the reach-and-grasp movement and simultaneously shift their gaze towards the self-chosen target. On the online trials, feedback on the *MOVE&LOOK* detections was provided through LEDs displayed on the targets. In the *LOOK* condition, participants solely performed the goal-directed saccade. **(B)** Source imaging results. The top and middle panels show the grand-average (*n* = 20) source space LFTD activity locked to the saccade onset for the *MOVE&LOOK* and the *LOOK* condition, respectively. Differences between both experimental conditions in the source space are shown in the bottom panel (only significantly different voxels are colored).

To eliminate the need for memorization tasks and a monitor to present visual cues, in Pereira et al. (2021) we have developed a paradigm that allowed for the online detection of self-initiated movements asynchronously in a more realistic scenario (Figure 3A). The largest body of studies that use EEG signals for movement detection involve tasks in which participants are typically requested to fixate their gaze at a specific point in the environment. These restrictions are imposed due to concerns with regard to eye-dipole-induced artifacts which can easily contaminate EEG signals in the low-frequency bands here analyzed. In Pereira et al. (2021), EEG signals were locked to the saccade onset as 20 non-disabled participants were instructed to also shift their gaze towards the movement target when they initiated the goal-directed reach-and-grasp. This strategy allowed us to obtain a time-locking point to extract relevant movement-related features, and further, it allowed us to incorporate gaze. Special care was taken regarding artifact attenuation (see “*Artifact Handling*” Section), and an additional control oculomotor task was introduced. In this control task, participants solely performed a goal-directed saccade.

There was a clear fronto-central negativity in both conditions, but the negativity was significantly stronger for the reach-and-grasp condition. This stronger negativity peaked shortly before the recorded movement onset. The source imaging results corresponding to the difference between conditions in Figure 3B (bottom panel) point to brain areas associated with the generation of MRCPs. Superimposed on the fronto-central negativity, a positive potential located primarily in parietal sites was observed in the channel space. In the source space, this activity emerged mainly from the superior parietal cortex in both conditions (Figure 3B, top and middle panels). These LFTD features were exploited in a hierarchical classification approach to detect the goal-directed reach-and-grasp movement online. A true positive rate of 54% was obtained (chance-level 12%) and an average of 1.7 FP/min was reported on pure rest, and 1.2 FP/min within the main blocks of the experiment.

The study in Pereira et al. (2021) opens doors to more realistic settings and ultimately to tasks which incorporate not only motor but also visual processing and their EEG correlates in the real world. Finally, it is important to mention that there are other aspects which cover the domains of both perceptual and movement-related decision making and are interesting to investigate as well. One of them is for instance the target selection process in goal-directed movements: deciding on a target is not a strictly motoric process, and on BCI training paradigms the targets are often externally-cued. However, movement targets in a real-life scenario are often defined internally [as in Pereira et al. (2021)]. We also have started studying these processes in Pereira et al. (2018).

### Non-invasive Movement Decoding

In goal-directed movements, the brain does not only integrate visual and sensory information about whether to initiate a movement but also transforms the multimodal information into downstream commands for an ensuing movement, and, as the movement is executed, adapts movement commands according to incoming feedback (Cisek and Kalaska, 2010). Electrophysiology studies in non-human primates (NHPs) and fMRI studies in humans have shown that the extrinsic sensory information is transformed into intrinsic movement commands along the dorsal stream in fronto-parietal networks for reaching and grasping (Georgopoulos et al., 1982; Kalaska et al., 1989; Battaglia-Mayer, 2019). Whether the intrinsic movement commands rather reflect kinetics, kinematics or synergies is an ongoing debate (Omrani et al., 2017).

In the non-invasive domain, LFTD signals have been reported to primarily encode information about executed movements (Gu et al., 2009; Jochumsen et al., 2013; Ofner et al., 2017; Schwarz et al., 2018). Following the vast majority of previous magnetoencephalographic (MEG)/EEG studies, we focused our analysis on decoding the kinematics of executed and attempted movements. Some groups reported that they could classify the direction of discrete, center-out upper-limb movements phase-locked to the movement onset (Waldert et al., 2008) or even decode the movement trajectories (Bradberry et al., 2010), while others reported the absence of directional information at the movement onset (Antelis et al., 2013). The different findings raised the question whether MRCPs, phase-locked to the onset of discrete movements, are modulated by movement direction.

Complementary to discrete movement tasks, a number of studies investigated the expression of directional information during continuous movements (Lv et al., 2010; Ofner and Müller-Putz, 2012; Kim et al., 2015) and volitional states (Kim et al., 2015; Ofner and Müller-Putz, 2015). The majority decoded positions and/or velocities with linear models from LFTD features (Robinson and Vinod, 2016). When decoding movements, the trajectories can be reconstructed by integrating decoded velocities (Bradberry et al., 2010; Ofner and Müller-Putz, 2012), by directly decoding positions (Ofner and Müller-Putz, 2012), or by decoding and merging both types of kinematic signals (Li et al., 2009). In the invasive domain, it was shown that the spiking activity of neurons in M1 carries information about both types of kinematic signals (Wang et al., 2007), and that the activity is preferentially tuned to velocities rather than positions (Paninski et al., 2004; Wang et al., 2007). Since spiking activity and EEG activity reflect fundamentally different spatial scales, it is not clear whether the tuning characteristics of M1 neurons transfer to EEG activity.

Although discrete, center-out reaching movements are well suited to study MRCPs, they are not suited to study the tuning characteristics of EEG activity to positions and velocities. In a center-out task, the position and velocity signals are tightly correlated, limiting the chance of identifying the position and/or velocity-related effects in the EEG activity. To address both research questions, we designed an experiment that involved center-out and continuous goal-directed movements.

**Figure 4:**
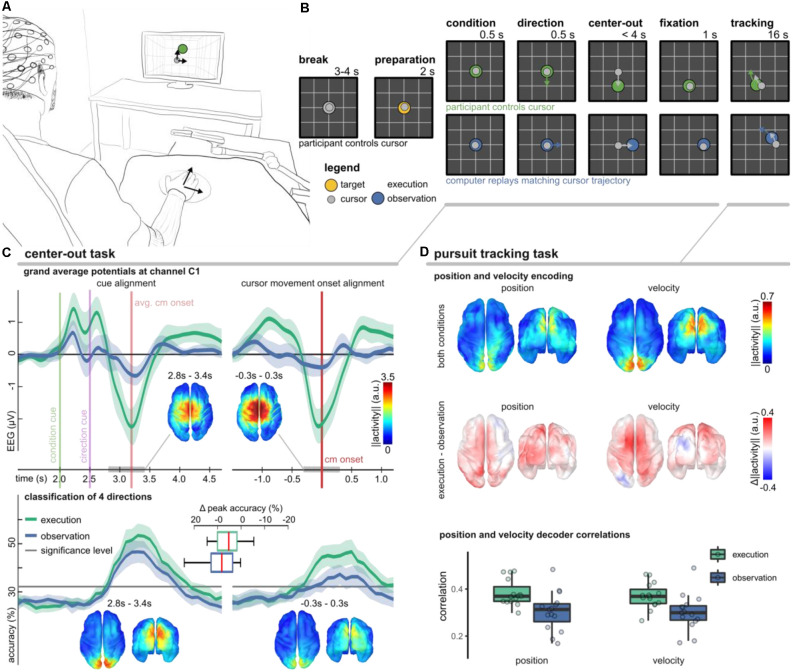
Non-invasive correlates of upper-limb movement kinematics adapted from Kobler et al. (2018); Kobler et al. (2020a); Kobler et al. (2020c). **(A)** Experimental setup. The participants’ right hand’s palm position was tracked on a 2D surface with a motion capture system (Leap Motion; Leap Motion Inc., USA); the hand movements were mapped to cursor movements in a virtual 2D workspace. **(B)** The trial-based paradigm considered two conditions—execution and observation. In execution condition trials, the participants controlled the cursor, while in observation condition trials, matching cursor movements were replayed. We considered two tasks. In a center-out task, a target moved for 0.5 s in one direction and stopped. After the cursor reached and maintained the target’s position for 1 s, the pursuit tracking task started. **(C)** Results of the center-out task. The plots in the top panel show the grand average potential at channel C1 for both conditions and two alignments (cues, cursor movement onset). Shaded areas indicate confidence intervals. Comparing the conditions, the grand average traces differed most around the movement onset. Source space plots visualize how the condition factor was encoded around the movement onset. Direction-related results are summarized in the bottom panel. It contains accuracy curves for both conditions and alignments and source space plots that visualize how the direction factor was encoded around the movement onset in either alignment. **(D)** Position, and velocity decoding results during the pursuit tracking task. The source space plots in the top summarize how the position (left) and velocity (right) were encoded in the LFTD features irrespective of the condition, while the plots below show the differences across conditions (execution − observation). Each source space plot depicts the average position or velocity-related activity within a 300 ms sliding window ([−0.3, 0.0] s). We used the same sliding window for a PLS-based Wiener filter decoder. The boxplots display correlations between the recorded and decoded cursor position and velocity trajectories. The significance levels for positions and velocities were at 0.12 and 0.10.

The experimental setup and trial-based paradigm as well as the main findings are summarized in Figure 4. We considered two conditions (execution, observation) to study different volitional states. In either condition, the participants were asked to fixate a target stimulus with their gaze in a 2D workspace. During execution condition trials they additionally controlled a cursor by moving their right arm. We used the center-out task to investigate whether MRCPs are modulated by movement direction (Kobler et al., 2020a) and a pursuit tracking task (PTT) to investigate the tuning characteristics of positions and velocities (Kobler et al., 2018).

In the execution condition, we observed a prominent MRCP around the movement onset (Figure 4C, top panel). As expected, the MRCP at the movement onset contributed to a successful classification of the experimental conditions (execution vs. observation; 78.2% classification accuracy). In Kobler et al. (2020a), we also observed significant classification accuracies for the movement direction (50.6% for four classes). However, if we aligned the data to the presentation of the cues rather than to the cursor movement onset, the movement direction could be detected with significantly higher accuracy (55.9%). The direction classifier accuracy curves are summarized in Figure 4C (bottom panel). Using a general linear model that considered the factors’ condition and direction for both types of alignments (cues, cursor movement onset), we could identify cortical networks encoding condition and direction-related information. We found a consistent representation of movement direction in the parieto-occipital cortex 300–400 ms after the direction cue irrespective of the condition. A consistent representation of movement direction in the parieto-occipital cortex agrees with reports of previous fMRI studies, which implicated this region in reaching and eye movements and showed that it has a retinotopic organization in humans (Fernandez-Ruiz et al., 2007; Fabbri et al., 2010; Magri et al., 2019). The EEG activity originating in the sensorimotor cortex encoded less information about the movement direction. Moreover, the encoded directional information in the sensorimotor cortex was less consistent across participants and specific to the execution condition. Combining the neurophysiology and classification results, the findings of this study suggest a stronger representation of movement direction in parieto-occipital areas phase-locked to the presentation of the cues rather than in sensorimotor areas phase-locked to the movement onset.

In every second trial, the center-out task was followed by the PTT. As in Paninski et al. (2004), we designed the target stimulus’ trajectories so that the position and velocity trajectories were decorrelated at lag 0 and independent across the two dimensions. In analogy to the center-out task, we also observed in the PTT that the parietal-occipital cortex carried significant directional information in either condition (Figure 4D, top). By contrasting the encoding strength of directional information between the conditions, we found that the PM and contralateral M1 encoded more information about the cursor velocity in the execution condition (Figure 4D, middle). The temporal tuning characteristics indicated that the activity led the cursor velocity by approximately 150 ms (Kobler et al., 2018). These observations in the EEG are in agreement with the tuning characteristics of the spiking activity of M1 neurons (Paninski et al., 2004). Offline, we used a partial least squares (PLS) regression-based Wiener filter to decode the cursor velocity trajectories from LFTD features of the past 300 ms, and obtained moderate correlations in execution (0.4) and observation (0.35) conditions (Figure 4D, bottom).

**Figure 5:**
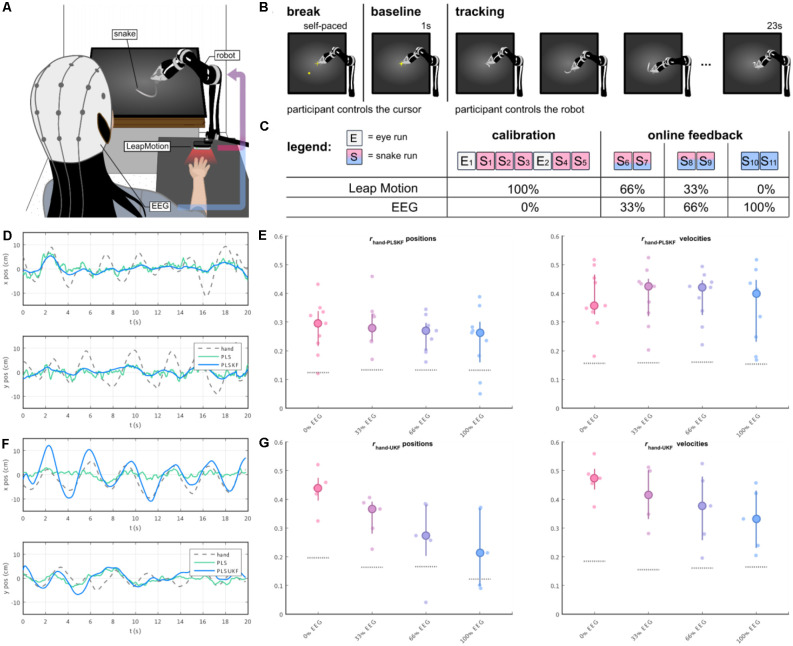
**(A)** The participants’ right-hand positions were recorded with a LeapMotion controller. During the experiment, participants were controlling a moving arm to track a moving object (i.e., the snake) on the screen. The control signal of the robotic arm was a mixture of hand kinematics (recorded by the LeapMotion) and EEG-based decoded trajectories, with a changing proportion over the course of the experiment. **(B)** Each trial started with a self-paced break, where the participants were in control of a cursor. As the participants wanted to start a new trial, they moved the cursor to the center of the screen, and held it still for 1 s (baseline). A moving trace was then displayed for 23 s, marking the beginning of the tracking period. **(C)** The experiment was divided into two parts, namely the calibration and the online part. The main experimental paradigm was implemented in the “snakeruns”, whose trials had the structure depicted in **(B)**. During the online part, the proportion of the EEG-based decoded trajectory was progressively increased every two snakeruns, first with 33%, 66% and up to the final condition of 100% EEG control. **(D,E)** Sample decoded trajectories and Pearson’s correlation r distributions between hand kinematics and PLSKF-decoded trajectories in the first online study (Mondini et al., 2020). **(F,G)** Sample decoded trajectories and Pearson’s correlation r distributions between hand kinematics and PLSUKF-decoded trajectories in the second online study (Martínez-Cagigal et al., 2020).

In a follow-up study (Mondini et al., 2020), we investigated the feasibility of continuously decoding voluntary hand/arm movement trajectories from the EEG, to achieve closed-loop online control of a robotic arm. The experimental setup is depicted in Figure 5A. The paradigm implemented a PTT, where the participants were asked to track a moving object on the screen (the “snake”, Figure 5A) by controlling a robotic arm. In the first part of the experiment, the participants performed some calibration runs with the robot fully controlled by their hand kinematics. After the EEG decoding model was fitted to the movement, the control signal for the robotic arm was gradually switched from kinematics- to EEG-based decoded trajectories, first with 33%, then 66%, up to a final condition of 100% EEG control (Figure 5C). PLS regression was once again used to decode several movement parameters (namely, the two-dimensional positions, velocities, and accelerations) from the EEG. To integrate the information from the different decoding models, we introduced a combined PLS and Kalman filtering approach, named PLSKF (Mondini et al., 2020). We obtained moderate yet overall significantly better than chance correlations between the hand kinematics and the PLSKF-decoded trajectories of (0.28, 0.29, 0.26, and 0.24) on average, for the (0%, 33%, 66%, and 100%) EEG control conditions, respectively (Figure 5E). For the sake of comparison, we simulated offline the correlations that would have been obtained with PLS regression alone. With respect to PLS regression, the PLSKF led to a stable correlation increase of Δr = 0.049 on average, demonstrating the successful integration of different decoding models. The level of robot control was above the chance level in all conditions, and participants finally reported feeling enough control to be able to improve with training, even in the 100% EEG condition. Despite the encouraging results, we found an amplitude mismatch between hand kinematics and decoded trajectories, which could not be fixed with the PLSKF model (Figure 5D).

More recently in Kobler et al. (2020c), we suggested that integrating information about non-directional kinematics (e.g., distance, speed) in the decoding model can alleviate the problem of amplitude mismatch. Recent studies suggest, indeed, that cortical signals not only carry information about movement direction (e.g., positions, velocities), but also information about movement amplitude (e.g., distance, or speed). This non-directional kinematic information has been found in both ECoG (Hammer et al., 2016) and MEG activity (Kobler et al., 2019a). Provided that distance and speed are nonlinearly related to position and velocities, the previously introduced PLSKF approach (Mondini et al., 2020) had to be extended to an Unscented Kalman Filter (UKF), which we denote PLSUKF here. In Kobler et al. (2020c), the EEG data from Kobler et al. (2018) and MEG data from Kobler et al. (2019a) were reanalyzed, to evaluate the performance of the PLSUKF with respect to both the PLS and the PLSKF, during both observed and executed movements. The correlations between the executed and the decoded trajectories were higher compared to the other algorithms, specifically 0.49 on average during execution and 0.36 during observation. In addition, the integration of non-directional kinematics in the decoding model could reduce the amplitude mismatch between recorded and decoded trajectories (Figure 5F, with respect to Figure 5D), thus overcoming the limitations of the previous study.

We tested how the results of the PLSUKF would translate to a closed-loop online scenario, recently in Martínez-Cagigal et al. (2020). The study implemented the same paradigm with the robotic arm as in Mondini et al. (2020) (Figures 5A–C), with the main difference of using a PLSUKF-based decoder. We obtained grand average correlations between hand kinematics and PLSUKF-based decoded trajectories of (0.43, 0.34, 0.27, 0.23) on average for the (0%, 33%, 66%, 100%) EEG control conditions (Figure 5G). Moreover, the PLSUKF could adjust the amplitude mismatch (Figure 5F), with a grand average amplitude ratio of 1.07 between recorded and decoded movements.

**Figure 6:**
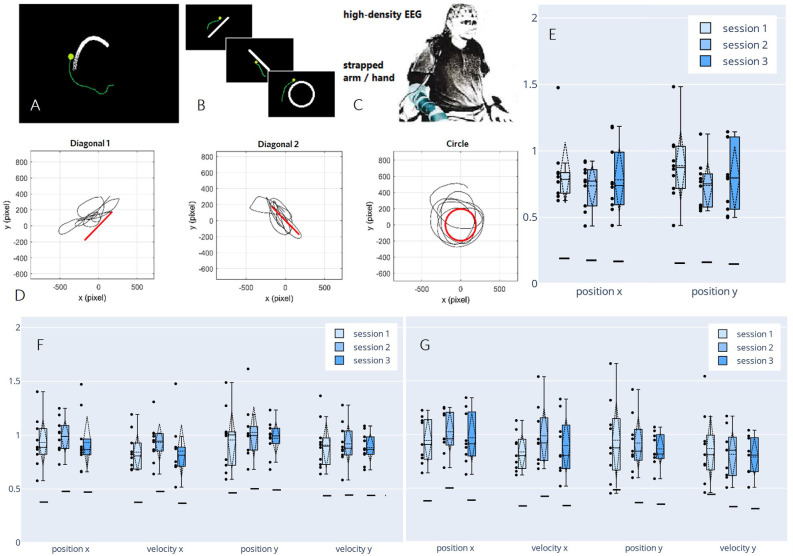
Paradigm,decoded trajectories, and grand average correlations adapted fromPulferer et al. (2021). **(A–C)** Paradigms, and experimentalsetup. Participants were asked to trace the object onscreen—the “snake” during snakeruns (**A**,tracking task) and static shapes during freeruns (**B**, tracingtask)—with their gaze while simultaneously attemptingmovement with the dominant hand/arm strapped to the chair as they sat in front of the screen **(C)**. Online feedback was delivered as a green dot. **(D)** Exemplary freerun shapes with corresponding EEG-decoded trajectories (P4, session 2). **(E–G)** Normalized (w.r.t. the mean correlation achieved in the calibration snakeruns of the respective session) correlations over sessions for freeruns (**E**: 100% EEG feedback) and snakeruns (**F**: 50% EEG feedback, **G**: 100% EEG feedback). Grand average correlations between EOG-decoded trajectories (freeruns) resp. snake (snakeruns) and EEG-decoded trajectories with standard deviations (dashed vertical lines and tails), single participant means (dots), median and 25th/75th percentiles (boxes), and median chance levels for all movement parameters.

Having demonstrated the feasibility of decoding continuous executed movement in non-disabled participants, we proposed a new paradigm in a follow-up study to advance our setup toward motor-impaired end-users. In a pilot study, we investigated the viability of decoding from attempted movement, mimicking the limited motor function a spinal cord injured person would experience by strapping each participant’s dominant arm to the chair he/she was seated on (Figure 6C). In the said pilot study, we not only observed correlations in a similar order of magnitude to the correlations achieved by decoding from actual movement, but we also found an increase in perceived performance towards the end of the session, as reported by the participant. Consecutively, we decided to investigate possible learning effects that may arise when training a BCI user multiple times on the same motor control task (Pulferer et al., 2021), which we now discuss in more detail.

In three sessions within 5 days per participant, the EEG of 10 non-disabled participants was recorded. The time frame was chosen to ensure that the participants could recuperate from the mental strain of the diverse tasks, yet still retain a clear recollection of prior sessions. Within two different paradigms—*snakeruns* originally described in Mondini et al. (2020) and Müller-Putz et al. (2021) and *freeruns* (Figures 6A,B)—the participants were asked to visually track a target or trace a fixed shape on a screen while simultaneously attempting a corresponding movement with their strapped arm as if wielding a computer mouse. As before, eye-dipole-induced artifacts were corrected as outlined in Section “Artifact Handling”. For each session, four calibration snakeruns (48 trials) using fake feedback (delayed snake) were performed for fitting the decoder, followed by three snakeruns (36 trials) each with first 50% and then 100% EEG feedback until finally, three freeruns (36 trials) using 100% EEG feedback were recorded (Figure 6D). Pearson’s correlation coefficient was again chosen as an evaluation metric, yielding the results shown in Figures 6E–G, which amount to grand average correlations across all movement parameters of 0.31 (0.02 SD), 0.32 (0.02 SD), and 0.30 (0.02 SD) for sessions 1–3 in both the online tracking tasks combined. These values range between corresponding results of 0.31 (0.08 SD) during an observation-only task, and 0.40 (0.06 SD) during executed movement in identical tracking tasks of slightly shorter duration (16 s) as reported previously in Kobler et al. (2020c). Due to a lack of ground truth trajectories (snake) in the freeruns, we estimated the decoder performance between the EEG-decoded and the eye movement inferred trajectories. Horizontal and vertical eye movements were estimated from electrooculographic (EOG) activity. To avoid variation due to differences in decoder performance from session to session, the correlations were normalized with respect to the mean correlation during the calibration runs in each respective session. In all feedback conditions and paradigms, we observed single participant means (dots) and hence grand averages (dashed horizontal lines) exclusively above chance level (solid horizontal lines). A slight though non-significant improvement from the first to the second session could be observed for all kinematic parameters in the 50% EEG feedback condition as well as for the parameters along the x-axis in the 100% EEG feedback condition, implying a positive effect of user training on the decoding performance, followed by performance degradation from the second to the third session. Additionally, we observed a decrease in performance with increasing time interval from decoder calibration; while the grand average correlations reached close to calibration correlation during the 50% EEG feedback snakeruns (Figure 6F), the performance decreases consecutively in the 100% EEG feedback snakeruns (Figure 6G) and reaches the lowest results during the freeruns (Figure 6E). A multitude of factors could potentially explain these observations. For example, nonstationarities in the EEG signals could have led to a degradation of the decoder performance over time. Alternatively, the users’ task engagement could have declined as they got accustomed to the paradigms and tasks. Furthermore, the intrinsic differences in the dynamics of both paradigms could explain why the performance of a model trained on the snake runs with fake feedback degrades when it is transferred to freeruns with different dynamics as well as to snakeruns where the decoder output modifies the task dynamics. As this issue is not limited to non-invasive decoding, established closed-loop control techniques from the invasive domain might aid in transferring open-loop calibrated models to closed-loop control (Gilja et al., 2012; Willett et al., 2018).

**Figure 7:**
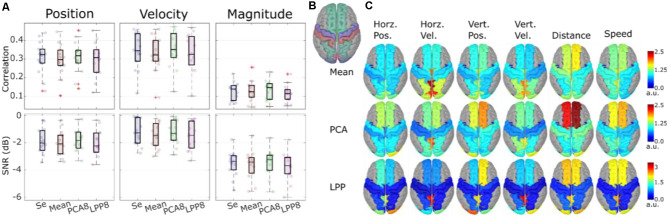
Theoverall results from the source-space decoding. Adapted from Srisrisawang and Müller-Putz (2021). **(A)** The decoding performance resulting from the sensor-space decoding approach (Sensor), and the source-space decoding approaches (Mean, PCA8, and LPP8). The average metrics were computed within the 0%–100% EEG. The horizontal and vertical of the directional kinematics were grouped into position and velocity. The non-directional kinematics were grouped into magnitude. **(B)** The defined regions of interest. **(C)** The decoding pattern. In the case of PCA and LPP, only the first component is shown.

We also investigated the possibility of performing the hand trajectory decoding in source space (Srisrisawang and Müller-Putz, 2021). To do so we based the processing steps on Martínez-Cagigal et al. (2020). We introduced an additional source-space transformation and extracted the activity of regions of interest (ROI). These additional steps were done *via* source imaging (Michel et al., 2004). First, we modeled 5,000 unconstrained current dipole sources (each source comprises three directional components) across the ICBM152 template head model with the boundary element method. Then, we solved the source localization problem using sLORETA *via* the brainstorm package (Tadel et al., 2011). We addressed the problem of a high number of dimensions in the source space *via* a two-fold dimensionality reduction approach. First, we defined ROIs according to the frontoparietal network identified in our previous studies (Kobler et al., 2018, 2019a, 2020a). The defined ROIs can be seen in Figure 7B. Second, we applied three different dimensionality reduction techniques to each directional source component of each ROI. The techniques were: averaging, principal component analysis (PCA), locality preserving projection (LPP; He and Niyog, 2003). For PCA and LPP, we empirically determined the number of components to be retained in each ROI as 8, due to saturation of the decoding performance. We then compared the decoding performance for the following cases: sensor-space decoding (Se), Mean, PCA8, LPP8.

Figure 7A shows the decoding performance in terms of correlation and SNR where each circle represents the corresponding performance metric of each subject. The gray lines connected between the median values were added to facilitate the comparison. To simplify the visualization, the horizontal and vertical components were grouped together into position and velocity and the distance and speed were grouped into magnitude. Generally, a similar range of correlation and SNR could be observed across all approaches (correlations: around 0.3 for position and velocity and around 0.1 for magnitude, SNRs: around −2 dB for position and velocity and −3 dB for magnitude). Mean and LPP8 indicated slightly lower correlations and SNRs than Se, while PCA8 showed a small improvement in terms of correlations for velocity and magnitude and in terms of SNRs for position and magnitude. Overall, we observed statistically significant differences only between LPP8 and PCA8. PCA8 showed on average an improvement in terms of correlations in comparison to Se by 0.0043 and in terms of SNRs by 0.0476 dB. The decoding patterns at time lag 0 ms (Figure 7C) revealed a strong dependence on the brain regions in the parieto-occipital cortex for velocity decoding as well as in the frontal regions in the distance and speed decoding. We concluded from these results that the source-space-based decoding is possible with similar performance as done in the sensor space.

### Grasp Representation

Electromyographic and kinematic information have been proposed as candidates for the neural representation of hand control (Ejaz et al., 2015; Leo et al., 2016). However, it remains unclear how these movement covariates are reflected in the EEG activity during different stages of grasping movements, such as hand-preshaping, reaching the final grasping posture, and holding.

In an exploratory study (Sburlea and Müller-Putz, 2018), we simultaneously acquired EEG, kinematic and electromyographic signals in 31 non-disabled human subjects while observing 33 different pictures of hand-object interaction and executing the grasps previously observed. Our study aims were three-fold. First, we investigated the relation between EEG and the behavioral covariates associated with the movement execution phase. Using representational similarity analysis, we found that EEG activity reflected different movement covariates in different stages of grasping. During the pre-shaping stage, centro-parietal EEG in the lower beta frequency band reflected the object’s shape and size, whereas, during the finalization and holding stages, contralateral parietal EEG in the mu frequency band reflected muscle activity. Second, we asked how the EEG patterns of static grasping observation relate to the behavioral covariates of movement execution (Sburlea and Müller-Putz, 2019). We found that the EEG representation of the observation phase in the mu and low beta frequency bands was correlated with the muscle representation during the execution, most strongly in the movement holding phase. This similarity indicates that when visually processing the hand-object interaction, we focus on the final grasping posture. Third, we investigated whether the muscle envelope of different grasping movements can be continuously predicted from LFTD EEG amplitudes using a filtering approach (Sburlea et al., 2021). We achieved higher prediction accuracy for intermediate grasps compared to power or precision grasps.

To confirm the hypothesis, derived from the previous study, which surmises that the shape of the objects is encoded in the brain patterns from parieto-occipital regions during hand preshaping, we conducted a new study (Sburlea et al., 2021). By separating properties of the objects from properties of grasping movements, we found a different spatial encoding of the grasp type and number of fingers (grasping movements) and the shape and size (intrinsic object properties) throughout the movement stages. Using LFTD EEG activity we reached significantly higher than chance level classification accuracy for both object properties and grasp types during the planning and execution of the movement. Therefore, this preferential time-wise encoding allows the decoding of object properties already from the observation stage, while the grasp type can also be accurately decoded also at the object release stage.

These findings contribute to the understanding of the temporal organization of neural grasping patterns and could inform the design of noninvasive neuroprosthetics and BCIs. Moreover, these findings allow us to gain a joint understanding of the relation between movement observation and execution, and represent a means to facilitate an intuitive control of neuroprostheses in motor-impaired individuals.

### Error-Related Potentials During Continuous Feedback

BCIs are still prone to errors when converting the user’s intentions into actions. The neural signature of error processing is known as error-related potential (ErrP) and can be measured using EEG when BCI users realize that the BCI committed a mistake (Ferrez and Millán, 2005). Initial research on the use of ErrPs on BCIs focused on the detection of ErrPs during discrete tasks, using a time-locked approach (Chavarriaga et al., 2014). More recently, the study of the continuous detection of ErrPs during continuous tasks emerged, using an asynchronous approach (Omedes et al., 2015; Spüler and Niethammer, 2015). Within the “Feel Your Reach” project, we focused on the study of the asynchronous detection of ErrPs during the continuous control of an end-effector.

**Figure 8:**
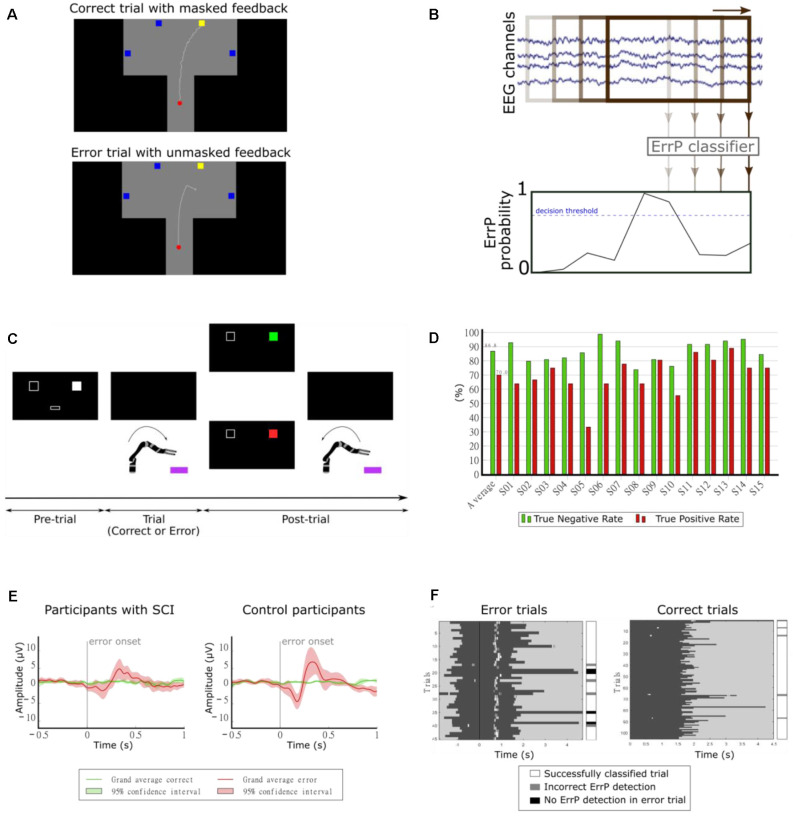
**(A)** Experimental paradigm. Participants controlled a cursor on the screen towards one of four target squares [adapted from Lopes-Dias et al. (2018)]. **(B)** Schematic representation of the asynchronous detection of ErrPs using a sliding window approach. **(C)** Experimental paradigm. Participants used their hand to control a robotic arm towards one of two boxes lying in front of the robot. Before each trial, a screen indicated the target box and after each trial, it presented feedback regarding the robot’s behavior [adapted from Lopes-Dias et al. (2019a)]. **(D)** Decoding results of the online part of the experiment [adapted from Lopes-Dias et al. (2019a)]. **(E)** Grand average correct and error signals at channel FCz for participants with SCI and control participants. The shaded areas represent the 95% confidence interval of the grand average curves [adapted from Lopes-Dias et al. (2021)]. **(F)** Decoding evaluation of one participant. Left: error trials, aligned to the error onset (black vertical line). Right: correct trials, aligned to the start of the trial. The dark gray areas represent the trials and the white marks within them represent the ErrP detections [adapted from Lopes-Dias et al. (2021)].

In our first study, we investigated the continuous detection of ErrPs in offline conditions (Lopes-Dias et al., 2017, 2018). We measured the EEG of 15 non-disabled participants while they controlled a cursor using a joystick towards one of four targets, as depicted in Figure 8A. There were two experimental conditions: one condition in which the cursor’s position was masked by the incorporation of a jitter component (masked feedback) and one condition with no jitter (unmasked feedback). Thirty percent of the trials were error trials. In these trials, the participants’ control of the cursor was interrupted at an unexpected moment during the trajectory. The remaining trials were named correct trials. We evaluated the time-locked classification of correct epochs against error epochs and obtained an average TPR of 81.8% and an average TNR of 96.4% (Lopes-Dias et al., 2018). Furthermore, we also investigated the asynchronous detection of ErrPs during the entire duration of the trials. To this end, we used a sliding window approach (Omedes et al., 2015), by continuously evaluating a window of the pre-recorded EEG, as depicted in Figure 8B. The output of the classifier was then transformed into the binary detection of ErrPs using a decision threshold.

The asynchronous detection of ErrPs was evaluated in a cross-validated manner and in a simulated online manner, using trial-based metrics that considered the entire duration of the trials. The asynchronous detection of ErrPs in the simulated online scenario yielded an average TPR of 64.5% and an average TNR of 84.0%.

Our following study tested the feasibility of asynchronously detecting ErrPs in an online condition and during the continuous control of a robotic arm. In this study, we measured the EEG of 15 non-disabled participants, who were instructed to move the robotic arm towards one of two targets in front of them, as depicted in Figure 8C. This experimental setup is intended to mimic a possible use of a BCI by an end-user (Lopes-Dias et al., 2019a). In 30% of the trials, the experimental protocol halted the participants’ control of the robot at an unexpected moment during the continuous movement towards the target (error trials). The remaining trials were named correct trials. This experiment comprised two distinct phases: offline calibration and online testing. During the offline calibration, we recorded the EEG signals of the participants while performing the task. These signals were used to train a personalized ErrP classifier for every participant, which was tested in the online part of the experiment. In this part, the participants could correct the errors elicited by the experiment, if an ErrP was detected in the participants’ real-time EEG signals. When this happened, the participants regained control of the robot and could reach the intended target. The asynchronous ErrP detection in the online part of the experiment, considering the entire trial duration, resulted in an average TPR of 70.0% and an average TNR of 86.8%, as depicted in Figure 8D.

The long duration of this experiment, due to its two phases, could hinder its transferability to BCI end-users. Hence, we decided to investigate a strategy to dismiss the offline calibration. Therefore, we proposed a generic classifier, which was trained with the EEG signals of 14 non-disabled participants and tested in a different participant. At a group level, the asynchronous ErrP detection with this classifier yielded a comparable performance to a personalized ErrP classifier (Lopes-Dias et al., 2019b).

Finally, we investigated the use of the generic classifier for the asynchronous detection of ErrPs in online conditions. In this study, we measured the EEG of eight participants with an SCI and eight non-disabled control participants, while they controlled a robotic arm, in an experimental setup similar to the previous experiment (Lopes-Dias et al., 2019a). The experiment required no offline calibration and the participants received feedback of their brain signals from the start of the experiment onwards. Participants with SCI displayed a more heterogeneous ErrP morphology than control participants (Lopes-Dias et al., 2021). At a group level, the grand average ErrP of the SCI group displayed lower peak amplitudes than the grand average ErrP of the control group, as depicted in Figure 8E. Nevertheless, participants in either group who displayed a clear ErrP morphology also obtained classification results above the chance level. Figure 8F illustrates the asynchronous ErrP detection in one control participant.

Summarizing, these works offer a strategy to asynchronously detect ErrPs without requiring prior offline calibration. This strategy was tested online with participants with SCI and with control participants. Moreover, these results can also promote the combination of ErrP detection with other control modalities when developing a BCI.

### Kinesthetic Feedback

**Figure 9:**
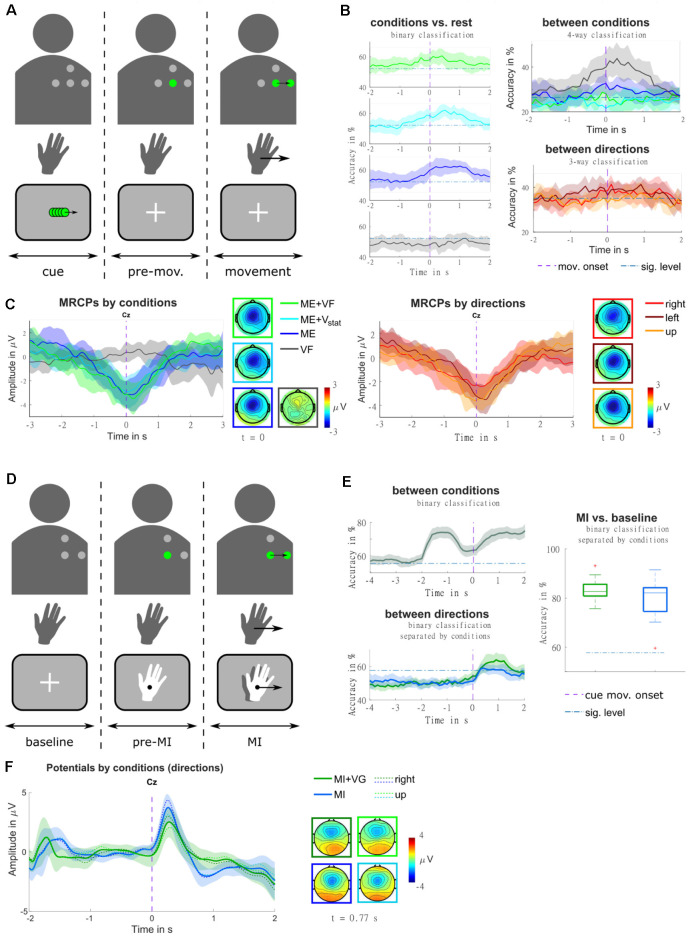
**(A)** Illustration of the trial structure, for an example trial with real-time feedback (condition ME+VF, direction right). In the top row, the four dots identify the layout of the vibrotactile actuators. They are presented in gray where they are off, and in green where they are active. The second row represents the hand movement. The movement period is indicated by an arrow in the movement direction. The bottom row contains a sketch of the visual input presented to the participant on a screen. At the beginning of each trial, a visual moving cue was presented, instructing the participant on the movement direction. Subsequently, the actuator representing the starting position was turned on, and a fixation cross appeared on the screen. Participants were instructed to stay in a relaxed state for a random delay period, and to initiate the movement in a self-paced manner [adapted from Hehenberger et al. (2020)]. **(B)** Classification accuracies are represented as grand averages and confidence intervals for each condition against rest, as well as for multi-class classification between conditions, and between directions. The purple dashed vertical lines mark the movement onset, and blue dash-dotted horizontal lines the threshold of significantly better-than-chance performance [adapted from Hehenberger et al. (2020)]. **(C)** MRCPs are represented as grand averages and confidence intervals, along with topographic representations at the time point of the movement onset. The averages were separated by conditions in the left panel, and by movement directions (movement trials only) in the right panel [adapted from Hehenberger et al. (2020)]. **(D)** Illustration of the trial structure, for an example trial with vibrotactile guidance (condition MI+VG, direction right), in a comparable fashion to panel a. Here, the second row represents the imagined hand movement, and the MI period is indicated by an arrow in the movement direction. At the beginning of each trial, a fixation cross was presented for a baseline period of two seconds, where participants were instructed to keep their gaze fixed and relax. Subsequently, the actuator representing the starting position was turned on, and the hand cue appeared. After a delay of two seconds, both the hand and the vibrotactile input started moving, guiding the participant’s imagined movement adapted from Hehenberger et al. (2021). **(E)** Classification accuracies for classification between conditions and between directions are represented as grand averages and confidence intervals, as well as MI vs. baseline represented as box plots of the grand average accuracies. Purple dashed vertical lines mark the cue movement onset, and blue dash-dotted horizontal lines the threshold of significantly better-than-chance performance [adapted from Hehenberger et al. (2021)] **(F)** Potentials are represented as grand averages and confidence intervals, along with topographic representations at the time point of the grand-average MRCP peak [adapted from Hehenberger et al. (2021)].

Our endeavors to provide artificial somatosensory feedback focus on transmitting kinesthetic information by non-invasive means. After screening the possibilities of several potential modalities, including non-contact ultrasonic acoustic radiation force, electrotactile stimulation, and vibrotactile stimulation *via* piezoelectric buzzers, magnetic transducers, and specialized electromagnetic vibrotactile actuators, we concluded that the latter would be the most suitable to our intention of stimulating the shoulder blade or upper back, where the tactile receptor density is relatively low. Specifically, we have conducted the following investigations with C-2 tactors (Engineering Acoustics Inc., Casselberry, USA), controlled by a custom device including an ARM Cortex M4 microcontroller (STMicroelectronics, Geneva, Switzerland). The tactors were attached to the inside of an elastic shirt (Figures 9A,D).

In order to realize spatially continuous feedback with a low number of discrete actuators, we tested stimulation patterns intended to evoke moving sensations in a small behavioral pilot study. Moving sensations can be achieved by exploiting tactile illusions resulting from imprecise tactile perception, both in the spatial and the temporal domain. When the skin is presented with two stimuli that are too close together to discern their locations, they are interpreted as a single “virtual” stimulus at a location in between (Alles, 1970). Furthermore, when a stimulus is followed by another at an interval too short for them to be perceived separately, the illusion of apparent tactile motion occurs (Sherrick and Rogers, 1966; Kirman, 1974; Israr and Poupyrev, 2011). In our pilot study (Hehenberger et al., 2019), patterns were computed such that a virtual stimulus would travel from one tactor to another, where the virtual stimulus was computed according to three different models (linear, logarithmic, power), regarding the relation of tactor intensities and desired stimulus location. Based on participant responses, we concluded that the power model most accurately maps the location of virtual stimuli. Our findings were in line with conclusions reached by Israr and Poupyrev (2011) and Luzhnica et al. (2017) for stationary stimuli.

In the context of “Feel Your Reach”, it is relevant to examine to which extent vibrotactile feedback would influence EEG signals, be it by introducing artifacts or by impacting sensorimotor processing due to the additional somatosensory input. In our first study in a non-disabled population regarding this concern (Hehenberger et al., 2020), we recorded four conditions and three movement directions in a center-out task, in order to get a broad overview. In the first condition (condition ME+VF), participants received real-time vibrotactile feedback of the hand position. In the second and third conditions, they were provided with static vibrations carrying no information (condition ME+V_stat_), and no stimulation (condition ME), respectively. In the fourth condition, they were instructed not to perform any movement while receiving sham feedback (condition VF). The movements were performed in a self-paced manner, following a cue indicating the movement direction (i.e., right, left, or up^1^). As depicted in Figure 9C, grand-average MRCPs exhibited a marginally larger peak in condition ME+VF, compared to condition ME, both shortly after the movement onset. In condition ME+V_stat_, the initial slope is similar to the feedback condition, but the MRCP peaks at the time point of the movement onset. This peak is weaker than in the other two movement conditions. The MRCP is localized centrally, with a slight lateralization to the contralateral side. In the two movement conditions containing vibrotactile stimulation (i.e., conditions ME+VF, ME+V_stat_), it slightly expands to the ipsilateral side. The presence of vibrotactile stimulation seemed to prompt 11 of 12 participants to initiate movements significantly earlier than in trials without stimulation. The three movement conditions could be classified against rest, based on LFTD features, with peak accuracies of 60%–65% (significantly above chance), while accuracies for the non-movement condition do not exceed the chance level. In a four-way classification, condition VF and condition ME could be discriminated at a within-class accuracy significantly above chance, while conditions ME+VF and ME+V_stat_ could not. The accuracies are shown in Figure 9B. When separating trials according to the three movement directions (movement trials only), regardless of condition, the grand average MRCP peak of movements to the right is considerably lower than for movements to the left or forward. These amplitude differences could potentially be attributed to differences in speed. Indeed, movements to the right were performed significantly faster in a majority of subjects. We hypothesize that these behavioral differences could result from differences in the difficulty of performing the movements. Three-way classification between movement directions yielded within-class accuracies narrowly exceeding the significance threshold above chance around the movement onset. According to Kobler et al. (2020a), the time window around the movement onset contains poorer directional information compared to the time where the direction is cued.

This issue was taken into account in the design of a follow-up study (Hehenberger et al., 2021), where we replaced the executed movement with motor imagery, and directly cued the initiation of the imagined center-out movement. This study included two conditions—MI with concurrent visual and vibrotactile guidance (condition MI+VG), and MI with visual guidance only (condition MI). Furthermore, we reduced the number of directions to two (right, up). The potentials (0.2–5 Hz) resulting from a superposition of evoked responses and MRCPs are depicted in Figure 9B, for the pre-MI and the MI period. The grand-average MRCP peak is more pronounced and less variable in condition MI, while the spatial profile is broader in condition MI+VG (Figure 9E). Classification between the two conditions based on LFTD features revealed that they are discriminable with peak accuracies of 70%–90%, both during the MI and the pre-MI period. The two directions could be classified with peak accuracies varying between approximately 60% and 80% during the MI period. On average, the accuracies are higher and more consistent in condition MI+VG. However, the individual peak accuracies are not statistically different (alpha = 0.05). Classification of the MI period against baseline (taking fixed windows from each trial) yields a higher average of accuracies for condition MI+VG (75%–90%) compared to condition MI (60%–95%), though the median is close to identical (Figure 9F).

### Artifact Handling

When reaching toward an object people orient their attention to the object. This process naturally unfolds in an overt fashion *via* saccades between various points of interest (Sailer et al., 2005). The saccades and other types of eye movements as well as blinks introduce electrophysiological sources commonly referred to as EOG. Consequently, electrodes at the scalp capture both the EEG and EOG. This mixture of EOG and EEG activity in the recorded channels can severely confound the experimental findings if the eye movements covary with the experimental variables (e.g., kinematics).

EEG and EOG are typically disentangled by regression or independent component analysis approaches (Urigüen and Garcia-Zapirain, 2015). In online experiments, regression approaches are common due to their simplicity. However, the standard regression methods either undercorrect the eye artifacts or remove a considerable amount of brain activity (Schlögl et al., 2007). We recently proposed a short paradigm to record approximately 5 min of data during specific eye movements (Kobler et al., 2017) and have used it in our recent offline (Kobler et al., 2018; Schwarz et al., 2020; Kobler et al., 2020a, c) and online (Martínez-Cagigal et al., 2020; Mondini et al., 2020; Pereira et al., 2021) studies. In Kobler et al. (2020b), we proposed a new algorithm denoted sparse generalized eye artifact subspace subtraction (SGEYESUB). Using M/EEG data of 69 participants, we found that SGEYESUB achieved state-of-the-art eye artifact correction and at the same time maintained resting brain activity as well as MRCPs and ErrPs. SGEYESUB removed on average 1.5 μV from resting activity and less than 0.5 μV from the ERPs, while the residual correlations of the EEG channels with the EOG activity were below 0.1. More interestingly, using the calibration data and SGEYESUB to attenuate eye artifacts in the EEG, we could detect cortical activity that encoded information about the kinematics during visuomotor and oculomotor tasks offline (Figure 4) and online (Figure 5).

Considering an online scenario as in Figure 5, it is also critical to detect high-variance artifacts such as drifts, electrode pops, and muscle artifacts. These artifacts can be picked up and amplified by a decoder and in turn, severely confound the feedback for the user. In Kobler et al. (2019b), we proposed a simple algorithm denoted high-variance electrode artifact removal (HEAR). HEAR monitors the variance of each channel and converts it into an artifact probability using a short period of resting data. As the artifact probability increases the algorithm interpolates the affected channels’ signal with the signal of neighboring channels. HEAR proved effective to correct single electrode pops and drifts, while it cannot correct multi-channel muscle artifacts. However, the method can still be used to detect them, giving the paradigm or experimenter the chance to react.

## Discussion

In this work, we review and demonstrate a broad spectrum of works that build the basis for an EEG-based framework which should enable people with cervical SCI to control a robotic arm to assist in their daily activities, by the analysis of non-invasive brain signals only. We have demonstrated that non-invasive BCIs can be used to detect goal directed upper-limb movements and that we are able to decode kinematics, i.e., position, velocity, distance, and speed in 2D space. After developing decoding strategies, we have reported on online movement decoding as well as on decoding of trajectories of movement attempts. Furthermore, we present novel results about the multi-modal representation of human grasping movements. These findings contribute to a better understanding of the dynamical organization of non-invasive cortical patterns during reaching and grasping stages. Also, we show that online continuous ErrP detection in a robot control scenario is feasible, even when a generic classifier trained with data from non-disabled participants was transferred to people with SCI. Finally, we started to work on kinesthetic feedback that in the future should provide the end-users with additional feedback about the movement and the generated or measured forces of the end effector, i.e., the robotic arm.

To further achieve a full robotic arm control, we need to combine the various components in a hybrid BCI composed of all the aforementioned systems. The various systems would then be active when needed. For instance, an asynchronous goal-directed movement detection based on MRCPs features would act as a gating function on the kinematic decoders’ output before it would be sent as a control signal to the robotic arm. The ErrP detector would asynchronously monitor brain signals for error occurrence during robotic arm control. Kinesthetic feedback about the robotic arm’s state will be presented simultaneously to support the integration and usefulness of the robotic arm to the end-user.

One important aspect of the usage of such a framework to control a robotic arm in daily life situations is that end-users must be allowed to use their eyes in a natural way. The reasons are manifold: (i) people with severe paralysis are used to using their eye movements massively; (ii) to reach and grasp an object naturally includes oculomotor control; and (iii) eye-hand coordination is reflected in specific brain areas (Culham and Valyear, 2006; Filimon et al., 2009; Gallivan and Culham, 2015) and can contribute to decoding performance. For this reason, we developed algorithms that remove EOG artifacts from EEG. In EEG systems this is absolutely necessary, while in implanted systems, e.g., in Collinger et al. (2013), Ajiboye et al. (2017), and Benabid et al. (2019) artifacts caused by eye movements or muscle activities play no role.

All studies so far have been carried out with non-disabled participants and therefore all of these principles and methodology need to be transferred to end-users with motor impairments. Some aspects have already been transferred. For instance, we could show that ErrPs can also be successfully decoded online in end-users with SCI during a continuous control task (Lopes-Dias et al., 2021). We have also successfully demonstrated that MRCPs are still detectable and decodable in a single-case study with a person with a complete cervical SCI (Muller-Putz et al., 2019; Ofner et al., 2019). Concerning trajectory decoding, we think that one can anticipate a baseline decoder accuracy in paralyzed persons based on the decoder performance during oculomotor tasks (no arm or finger movements) in non-disabled persons, as explored in Kobler et al. (2018), Kobler et al. (2020a), and Kobler et al. (2020c). If a movement attempt strategy rather than mere observation is used, a similar performance to executed movements in non-disabled persons might be within reach (Vargas-Irwin et al., 2018; Rastogi et al., 2020).

One big challenge in BCI research is to minimize or even remove calibration time, which is usually needed before online experiments in non-invasive as well as invasive BCIs (Collinger et al., 2013; Ajiboye et al., 2017; Mondini et al., 2020). This is particularly challenging in complex paradigms that involve the decoding of multiple cognitive and motor functions. Simpler and more controlled setups have been shown to elicit more robust brain patterns and lead to a reduction in calibration time (Scherer et al., 2008). However, currently, when we want to study, e.g., learning effects of our framework, too long calibration can already decrease the motivation of participants (Pulferer et al., 2021). In one study we have shown that an ErrP asynchronous classifier trained on existing data of non-disabled participants could be successfully transferred to a population with SCI (Lopes-Dias et al., 2021). With the latest methodologies, e.g., with deep learning approaches, a session-to-session and even user-to-user transfer could overcome the necessity or at least drastically reduce within-session model calibration (cf. Wu et al., 2020; Huggins et al., 2021). Whether improvements on the feature engineering or machine learning side might be sufficient is not easy to predict. However, hybrid BCI solutions and intelligent shared control approaches that make use of external sensors could be a future option.

Even though we see no significant differences in decoding performance at the single session level (Srisrisawang and Müller-Putz, 2021), the transfer learning aspect of the source-space decoding approach might be interesting to investigate further as the specific anatomical information will be attenuated due to the projection into the source space. Then, the source-space signals might represent a common space (either across sessions or across participants). Alternatively, the domain information (session, user) could be used to learn data-driven models that either find a common shared subspace (Samek et al., 2012; Özdenizci et al., 2020), align data from different domains (Morioka et al., 2015; Dyer et al., 2017; Farshchian et al., 2019), or even utilize more robust metrics (Sabbagh et al., 2020; Kobler et al., 2021).

Overall, by proposing separate BCIs that each concern single necessary steps in a goal-directed movement task, we were able to address as well as resolve a set of major concerns in current BCI research. Including the identification of movement goals, artifact attenuation, online error detection, as well as kinesthetic feedback, we were able to show that in principle it is possible to reconstruct the kinematics of a continuous movement, which is an important step forward regarding natural control of end effectors for end-users compared to classical center-out tasks. However, it is not clear how meaningful movements can be realized, e.g., when from a starting position a user wants to reach towards a certain goal, perform a specific target-interaction there, i.e., grasp or release, and move to another place in space. EEG-based work in this direction is already done compare (Meng et al., 2016), however, not in a natural way, as we envision it. This is a future topic for investigations.

## Conclusion and Outlook

This work presents a first review of the work done in the project “Feel Your Reach” towards natural decoding of reaching and grasping based on non-invasive EEG. However, there is still work to be done to translate this into real life settings.

We believe that with the EEG enough information can be retrieved to successfully decode hand and arm movements to create control signals for artificial arms providing similar degrees of freedom as a human arm. We have provided the first evidence that in general, this can work on non-disabled populations, however, performance improvements are mandatory. We did not show 3D-movement decoding yet and need to get more experience and evidence from online studies. While we have started to include end-users, real evidence is still missing that people with tetraplegia due to an SCI at the cervical level above C4, with a total loss of arm and hand function, can benefit from such a system. Are brain patterns still observable after longer times since injury? Does the complexity of eye-hand coordination still work sufficiently? Can end-users be trained to use such a system? Evidence from works with implantable BCIs is given, however, the proof with non-invasive EEG still needs to be shown.

## Author Contributions

GRM-P had the general idea of the “Feel Your Reach” Project. All other authors contributed with their ideas and experience to the single article reviewed in this work. All authors contributed to the article and approved the submitted version.

## Conflict of Interest

The authors declare that the research was conducted in the absence of any commercial or financial relationships that could be construed as a potential conflict of interest.

## Publisher’s Note

All claims expressed in this article are solely those of the authors and do not necessarily represent those of their affiliated organizations, or those of the publisher, the editors and the reviewers. Any product that may be evaluated in this article, or claim that may be made by its manufacturer, is not guaranteed or endorsed by the publisher.

## Footnotes

^1^In “up” trials, the cue on the screen as well as the vibrotactile feedback on the back moved upward, while the movement was performed forward.
